# Contributions to Practical Medicine

**Published:** 1892-03

**Authors:** 


*Contributions to Practical Medicine.* By Sir JAMES SAWYER, Knt. Second Edition. Pp. 201. Birmingham: Cornish Brothers. 1891.

This book contains a series of disconnected essays on subjects which have been of interest, and have given repute, to the author; they were for the most part written many years ago, are now altogether out of date, and are not of sufficient clinical value to justify the present republication.

The essay on phthisical laryngitis, written in 1875, is said to have been "since revised and entirely rewritten;" and yet the author's observations lead him to conclude "that the local lesions in the larynx in phthisis are inflammatory, and unconnected with truly tubercular processes." Surely the author has never seen a section of laryngeal mucous membrane with the tissues infiltrated to a considerable depth with the specific products of tubercular action and crowded with bacilli.

The treatment of phthisical laryngitis, written in 1883, and professedly rewritten up to date, contains no reference to the use of cocaine, menthol, or lactic acid. The essay cannot be looked upon as a trustworthy guide, and is not worthy of serious criticism.

The essays on intestinal obstruction, pulmonary accentuation, floating kidney, and the treatment of gastralgia, give the views of an experienced observer in these subjects and are well worthy of perusal, but on the whole we think that the author's reputation will not be enhanced by a volume which needs in its preface an apology for incompleteness on the ground of pressure of daily clinical work.

